# Meta-analysis of cortical thickness abnormalities in medication-free patients with major depressive disorder

**DOI:** 10.1038/s41386-019-0563-9

**Published:** 2019-11-06

**Authors:** Qian Li, Youjin Zhao, Ziqi Chen, Jingyi Long, Jing Dai, Xiaoqi Huang, Su Lui, Joaquim Radua, Eduard Vieta, Graham J. Kemp, John A. Sweeney, Fei Li, Qiyong Gong

**Affiliations:** 1 Huaxi MR Research Center (HMRRC), Functional and Molecular Imaging Key Laboratory of Sichuan Province, Department of Radiology, West China Hospital of Sichuan University, Chengdu, Sichuan 610041 P. R. China; 2 Psychoradiology Research Unit of Chinese Academy of Medical Sciences (2018RU011), West China Hospital of Sichuan University, Chengdu, 610041 China; 3 Department of Psychoradiology, The Fourth People’s Hospital of Chengdu, Chengdu, China; 4 Institut d’Investigacions Biomèdiques August Pi i Sunyer (IDIBAPS), Mental Health Research Networking Center (CIBERSAM), Barcelona, Spain; 5 Centre for Psychiatric Research and Education, Department of Clinical Neuroscience, Karolinska Institutet, Stockholm, Sweden; 6 Department of Psychosis Studies, Institute of Psychiatry, Psychology and Neuroscience, King’s College London, London, UK; 7 Barcelona Bipolar Disorders and Depressive Unit, Hospital Clinic, Institute of Neurosciences, University of Barcelona, Barcelona, Spain; 8 Liverpool Magnetic Resonance Imaging Centre (LiMRIC) and Institute of Ageing and Chronic Disease, University of Liverpool, Liverpool, UK; 9 Department of Psychiatry, University of Cincinnati, Cincinnati, OH USA

**Keywords:** Diagnostic markers, Depression

## Abstract

Alterations in cortical thickness have been identified in major depressive disorder (MDD), but findings have been variable and inconsistent. To date, no reliable tools have been available for the meta-analysis of surface-based morphometric (SBM) studies to effectively characterize what has been learned in previous studies, and drug treatments may have differentially impacted findings. We conducted a comprehensive meta-analysis of magnetic resonance imaging (MRI) studies that explored cortical thickness in medication-free patients with MDD, using a newly developed meta-analytic mask compatible with seed-based *d* mapping (SDM) meta-analytic software. We performed the meta-regression to explore the effects of demographics and clinical characteristics on variation in cortical thickness in MDD. Fifteen studies describing 529 patients and 586 healthy controls (HCs) were included. Medication-free patients with MDD, relative to HCs, showed a complex pattern of increased cortical thickness in some areas (posterior cingulate cortex, ventromedial prefrontal cortex, and anterior cingulate cortex) and decreased cortical thickness in others (gyrus rectus, orbital segment of the superior frontal gyrus, and middle temporal gyrus). Most findings in the whole sample analysis were confirmed in a meta-analysis of studies recruiting medication-naive patients. Using the new mask specifically developed for SBM studies, this SDM meta-analysis provides evidence for regional cortical thickness alterations in MDD, mainly involving increased cortical thickness in the default mode network and decreased cortical thickness in the orbitofrontal and temporal cortex.

## Introduction

Major depressive disorder (MDD) is a major cause of disability and contributor to the global burden of disease affecting more than 300 million people worldwide [1, 2]. Despite its prevalence and disability, its neurobiological mechanisms remain incompletely understood. Magnetic resonance imaging (MRI) research has a significantly advanced understanding of brain changes associated with depression [3]. Many advances in clinical brain imaging research in recent years have been made possible by improvements in the measurement of distinct aspects of brain anatomy and function. In particular, advances in the measurement of cortical thickness, which reflects the size, arrangement, and density of neurons, nerve fibers and neuroglia, now can be performed with minimal partial volume effects that complicate brain volume estimates [4]. Advanced automated surface-based morphometric (SBM) methods, such as those available in FreeSurfer software, provide accurate models of the gray/white matter boundary and the pial surface of the cerebral cortex, measuring cortical thickness as the shortest distance between the two surfaces [5]. Cortical thickness abnormalities can be especially sensitive to regional disease-specific effects, including both neuroinflammation and other factors that can increase cortical thickness, and decreases in cortical thickness resulting from factors such as exuberant synaptic pruning and other causes of neuropil reduction [4, 6].

Studies of cortical thickness in MDD have had variable results. Some have reported increased cortical thickness in patients with MDD compared with healthy controls (HCs) in the orbitofrontal cortex (OFC) [7, 8], rostral middle frontal gyrus [7], superior frontal gyrus [8], parietal cortex [9], temporal cortex [10, 11], cingulate cortex [7, 9], occipital cortex [12], and insula [11]. Other studies have reported decreased cortical thickness in MDD in the middle frontal gyrus as well as OFC [13, 14], parietal cortex [15], temporal cortex [9, 16], occipital cortex [13], insula [11], anterior cingulate cortex (ACC), and parahippocampus [17]; some studies have reported no cortical thickness differences in these regions [18, 19]. These discrepancies may be explained by study differences in sample size, patient characteristics, clinical symptom severity, medication status, and image acquisition and processing protocols. By controlling for these, meta-analysis can help identify replicable and the most prominent cortical thickness alterations in MDD.

Two meta-analyses have examined cortical thickness variation in MDD. The Enhancing Neuro Imaging Genetics through Meta-Analysis (ENIGMA) consortium used an individual participant data-based approach to analyze cortical thickness data from 2148 patients with MDD and 7957 HCs in 20 international groups, synthesizing data across participating sites rather than from published literature [20]. Suh and colleagues used seed-based *d* mapping (SDM) software (www.sdmproject.com) for systematic quantitative comparison and synthesis of published studies, including articles from outside the ENIGMA consortium [21].

The inclusion of medicated patients may have biased the results for the two studies. In addition, the ENIGMA consortium focused on cortical thickness in regions of interest rather than every vertex of the cortex. In the absence of any specialized meta-analysis tool for SBM, Suh et al. used the gray-matter mask designed for voxel-based structural studies [21]. Cortical gray-matter volume is a function both of cortical thickness and surface area and in practice is more strongly correlated with the latter [4]. Cortical thickness is considered a heritable and relatively stable structural brain characteristic distinct from gray-matter volume [22]. SDM includes masks for different brain tissues [23], but the previously available gray-matter mask in SDM includes all gray matter (i.e., cortical and subcortical). If a meta-analysis of cortical thickness performed using the gray-matter mask in SDM, there would be an incongruence between the mask of the original studies (limited to cortical gray matter) and the mask of the meta-analysis, which could have an effect on cluster identification at cortical-subcortical boundaries and on significance testing given the different amount of brain tissue involved in analyses. Specifically, in the test of convergence using the gray-matter mask, the addition of voxels with artificially null effect size (the subcortical gray matter) could lead to the imprecise estimations of the statistical significance and cluster number in the cortical gray matter. Given these limitations, there has been a need to optimize SDM software for meta-analyzing cortical thickness studies. A new mask is needed specifically for studies of cortical thickness, making the SDM output optimized for meta-analyzing cortical thickness results from FreeSurfer.

Therefore, we set out to create a new meta-analytic mask in SDM to integrate the cortical thickness results of SBM studies in order to identify cortical thickness abnormalities in MDD across studies. We restricted our analysis to medication-free patients to reduce the potential impact of current/recent drug treatment on brain morphological changes. We hypothesized that: (i) compared with controls, medication-free patients with MDD would demonstrate cortical thickness alterations in OFC and ACC; and (ii) the identified altered cortical thickness would be associated with the severity of clinical features.

## Materials and methods

### Search strategy and selection criteria

The current meta-analysis was peformed according to the Preferred Reporting Items for Systematic reviews and Meta-Analyses guidelines (Table S1) [24]. The details of the search strategy and selection criteria are provided in Supplementary Methods. Two of us (Q.L. and Y.J.Z.) independently conducted the literature search. The results of these two searches were compared, and any inconsistencies were discussed and a consensus decision was reached about the appropriateness of the study for this meta-analysis.

### SDM meta-analysis

**Fig. 1:**
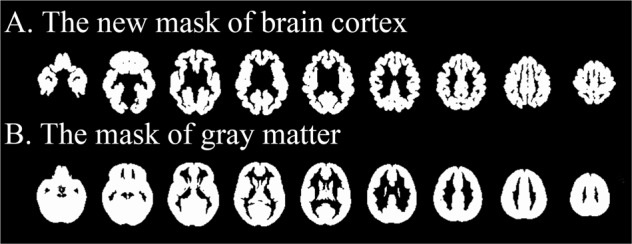
The demonstration of the newly created mask and the mask of gray matter previously available in SDM software. The demonstration of the newly created mask special for meta-analyzing the surface-based morphometric studies in the present study (**a**) and the existing mask of gray matter for meta-analyzing voxel-based structural studies in SDM software (**b**)

Meta-analysis was conducted in SDM software (version 5.15), which has been used to meta-analyse MRI data acquired from patients with psychiatric disorders including obsessive-compulsive disorder [25], schizophrenia [26], and autism spectrum disorder [27]. When conducting a meta-analysis, it is necessary to select an appropriate mask (e.g., gray matter, white matter, TBSS) to restrict the signed differential map created for each included study. As the existing gray-matter mask was not optimized for SBM studies, our first step was to adapt SDM by creating a new one (Fig. 1). The steps in this process were (1) resampling of FreeSurfer left and right hemisphere surface masks into volumes using the FreeSurfer command “mri_surf2vol”; (2) conversion of the volumes to NIfTI format with the FreeSurfer command “mri_convert”; (3) combining the two volumes (left and right hemisphere) into a single volume with the FSL command “fslmaths -add”; (4) performing a 12 degree-of-freedom affine transformation of the volume from FreeSurfer space to FSL-MNI space using the FSL commands “flirt -applyxfm” and “fslmaths -thr 0.01 -bin”; and (5) application of a narrow smoothing and binarization to make the mask slightly thicker and smoother (to avoid that it includes isolated voxels) with the FSL command “fslmaths -s 0.15 -bin”. In the pre-processing of the present meta-analysis, we found that nearly all (12 out of 13) of the maximum and minimum peak coordinates of the effect-size signed map of each included study were encompassed in the created cortical mask.

Details of quality assessment (Table S2), data recording, SDM method of meta-analysis, jackknife, heterogeneity, publication bias analysis, and meta-regression are presented in supplementary methods.

## Results

### Included studies and sample characteristics

**Fig. 2:**
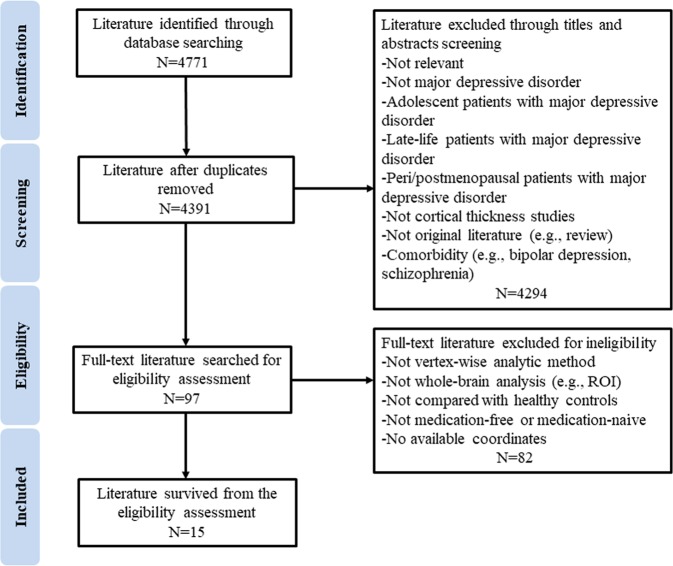
Flowchart of literature search and selection criteria. Search terms were (depression OR depressive disorder OR unipolar depression) AND (cortical thickness OR thickness)

Figure 2 shows the flowchart of the literature search and eligibility assessment. The final sample of 15 studies reported 529 medication-free patients with MDD (mean age 37.9 years) and 586 HCs (mean age 35.8 years); the meta-analysis incorporated 47 peak coordinates extracted from 11 of those studies [7, 9, 11–17, 28, 29]. Four studies were included in analyses that found no cortical thickness differences between patients and HCs [10, 18, 19, 30]. No study used overlapping patient samples. One study reported the coordinates of the peak on an inflated brain [29], so we converted the reported vertex numbers into the corresponding MNI coordinates that SDM requires, allowing consistent localization of alterations in cortical thickness for meta-analysis.

Table 1 shows the demographic and clinical characteristics from studies included in the meta-analysis. All participants provided written informed consent. Among the 15 studies, 7 recruited 224 drug-naive patients [7, 9, 13, 15, 19, 28, 29], while 4 studies included all off-medication but previously treated patients [10, 11, 18, 30], and 4 studies recruited samples combining  off-medication and drug-naive patients [12, 14, 16, 17]. The mean duration of illness was less in the drug-naive patients than the off-medication patients (1.0 years vs 9.2 years). The minimum time off medication in the studies of patients off medication included in our current study was at least 1 week (1 week, 1 study; 2 weeks, 1 study; 1 month, 1 study; 6 weeks, 1 study; 2 months, 1 study; and 6 months, 1 study) according to the six studies that reported the duration of the medication-free period before scanning [10, 11, 14, 16, 18, 30]. We considered all of these patients together as “medication-free” in our primary analysis.

**Table 1:** Demographic and clinical characteristics of participants in the 15 included studies

Study	Number (female)			Age at study, y			Age of onset, y	Illness duration, y	Mean number of episodes	Statistic of cortical thickness (correction)	Score of severity (scale type)	Medication status	Scanner field strength (Tesla)	Voxel size (mm^3^)
	MDD	HCs	*P* value for sex	MDD	HCs	*P* value for age								
Han et al. [19]	20 (15)	22 (15)	0.63	42.7	43.7	0.81	NA	0.4	1.8	*P* < 0.05 (MCS)	19 (HDRS-17)	All naive	3.0	1.0 × 1.0 × 1.0
Kakeda et al. [13]	40 (20)	47 (13)	0.05	46.6	40.7	0.06	NA	0.5	1.0	*P* < 0.05 (MCS)	22 (HDRS-17)	All naive	3.0	0.9 × 0.9 × 1.2
Lan et al. [30]	56 (32)	54 (26)	0.65	36.9	31.8	0.06	23.4	13.6	NA	*P* < 0.05 (MCS)	24 (HDRS-17)	All Untreated > 2 weeks	3.0	1.0 × 1.0 × 1.0
Liu et al. [28]	30 (13)	41 (13)	0.32	44.9	41.2	0.22	NA	NA	1.0	*P* < 0.05 (MCS)	21 (HDRS-17)	All naive	3.0	0.9 × 0.9 × 1.2
Na et al. [29]	45 (34)	72 (51)	0.31	41.6	40.7	0.35	NA	2.5	1.8	*P* < 0.05 (MCS)	20 (HDRS-17)	All naive	3.0	1.0 × 1.0 × 1.0
Niu et al. [16]	36 (19)	30 (13)	0.68	29.1	27.8	0.78	24.9	3.5	1.8	*P* < 0.05 (MCS)	27 (HDRS-24)	Untreated > 6 months (*n* = 17) Naive (*n* = 19)	3.0	NA
Peng et al. [9]	16 (9)	16 (9)	1.00	34.4	33.9	0.77	33.3	0.2	1.0	*P* < 0.05 (FDR)	31 (HDRS-24)	All naive	3.0	0.9375 × 0.9375 × 1.0000
Qiu et al. [7]	46 (33)	46 (33)	1.00	34.9	35.4	0.58	NA	0.4	1.0	*P* < 0.05 (FDR)	23 (HDRS-17)	All naive	3.0	NA
Späti et al. [14]	21 (10)	35 (20)	0.49	36.6	32.7	> 0.2	NA	NA	6.0	*P* < 0.05 (MCS)	26 (BDI)	Untreated > 6 weeks (*n* = 5) Naive (*n* = 16)	3.0	0.94 × 0.94 × 1.00
Taylor et al. [18]	74 (52)	91 (56)	0.24	36.4	29.9	NA^a^	NA	5.9	3.1	*P* < 0.05 (MCS)^b^	24 (MADRS)	All Untreated > 1 month	3.0	0.9 × 0.9 × 1.2
van et al. [12]	40 (27)	31 (19)	0.95	35.0	34.7	0.90	33.6	1.2	1.0	*P* < 0.05 (FDR)	13 (HDRS-17)	Untreated (*n* = 20)^c^/naive (*n* = 20)	1.5	1.0 × 1.0 × 1.0
van et al. [10]	23 (15)	22 (14)	0.91	50.7	50.8	0.98	40.8	NA	2.8	*P* < 0.05 (MCS)	22 (HDRS-17)	All Untreated > 1 week	1.5	1.0 × 1.0 × 1.0
Wagner et al. [17]	30 (25)	30 (25)	1.00	37.6	35.1	NA^d^	31.6	6.0	1.3	*P* < 0.05 (MCS)	25 (HDRS-21)	Untreated (*n* = 23)^c^/naive (*n* = 7)	1.5	1.0 × 1.0 × 1.0
Yang et al. [15]	27 (13)	27 (13)	1.00	28.6	28.7	0.07	27.8	0.8	1.0	*P* < 0.001 (Uncorr)	28 (HDRS-24)	All naive	3.0	1.0 × 1.0 × 1.0
Zorlu et al. [11]	25 (15)	22 (14)	0.71	36.7	38.5	0.45	30.6	NA	1.7	*P* < 0.05 (MCS)	26 (HDRS-17)	All Untreated > 2 months	1.5	1.0 × 1.0 × 1.0

*BDI* Beck Depression Inventory, *FDR* false discovery rate, *HCs* healthy controls, *HDRS-17/21/24* 17/21/24-item Hamilton Depression Rating Scale, *MADRS* Montgomery–Asberg Depression Rating Scale, *MCS* Monte Carlo simulations, *MDD* major depression disorder, *NA* not available, *Uncorr* uncorrected, *y* year

^a^Although this study did not demonstrate the result of age compared between all the patients (both without and with thoughts of death) and controls, age was controlled as a covariate for all the group comparisons of cortical thickness

^b^The author confirmed that there were no statistically significant differences in cortical thickness between the control group and the entire MDD group by email

^c^These studies did not report the period of the untreated state

^d^This study did not report the *P* values of mean age, but they stated no differences in age between patients with MDD and HCs

Three studies reported that some patients had comorbid psychiatric disorders (primarily anxiety disorder) [14, 15, 30], while the other 12 reported recruiting only patients without comorbidities. In 14 studies the threshold was corrected for multiple comparisons, but 1 study used an uncorrected threshold [15]. We also conducted separate subgroup meta-analyses in the 12 studies with 425 non-comorbid patients and 14 studies with threshold correction for comparison purposes.

The mean age of patients and the percentage of female patients were available in all 15 studies. In our meta-analysis, there was no statistically significant difference between patients with MDD (male, 197; female, 332) and HCs (male, 252; female, 334) in gender ratios (*P* = 0.05). Thirteen out of the 15 included studies reported the *P* values for the comparison of age between groups without statistically significant differences [7, 9–16, 19, 28–30]. For the two studies without the *P* values for age comparisons, Wagner et al. stated there were no differences in age between patients with MDD and HCs in their study without reporting the specific *P* value [17]. Taylor et al. recruited three groups in their study with depressed patients without and with thoughts of death and healthy controls, and they reported a statistically significant difference in age among the three groups (*P* < 0.0001) [18]. Although Taylor et al. did not demonstrate the result of age compared between all the patients (both without and with thoughts of death) and controls, age was controlled as a covariate for all the group comparisons of cortical thickness [18].

The age at onset and illness duration of patients were available for 7 studies [9–12, 16, 17, 30] and 11 studies [7, 9, 12, 13, 15–19, 29, 30], respectively. The mean number of episodes of illness in currently medication-free patients with MDD in 14 studies was 1.86, but unreported and unavailable in 1 study [30]. To evaluate illness severity at the time of testing of patients with MDD, 9 studies [7, 10–13, 19, 28–30], 1 study [17], and 3 studies [9, 15, 16] used the 17-, 21-, and 24-item versions of the Hamilton Depression Rating Scale (HDRS), respectively. One study used the Beck Depression Inventory [14], and 1 study used the Montgomery–Asberg Depression Rating Scale [18]. Based on the recommendation that meta-regression requires at least 9 studies [25], we explored by meta-regression the association with cortical thickness of age (15 studies), percentage of female patients (15 studies), illness duration (11 studies), number of episodes (14 studies), severity of illness (9 studies with 17-item HDRS).

### SDM meta-analysis

Pooling all 15 studies included in the meta-analysis, compared with HCs, medication-free patients with MDD showed increased cortical thickness in the left posterior cingulate cortex (PCC) (*Z* = 1.113, *P* = 0.00135) and right ventromedial prefrontal cortex (vmPFC) extending to left ACC (*Z* = 1.178, *P* = 0.00087), and decreased cortical thickness (cortical thinning) in the left gyrus rectus (*Z* = −1.412, *P* = 0.00027), left orbital segment of superior frontal gyrus (oSFG) (*Z* = −1.162, *P* = 0.00132), and right middle temporal gyrus (MTG) (*Z* = −1.144, *P* = 0.00146) (Table 2 and Fig. 2). The subgroup meta-analysis results in studies with non-comorbid patients and with threshold correction (Tables S3 and S4) were highly consistent with the pooled findings (Table 2). Most of the results were consistent between the medication-naive subgroup meta-analysis and the pooled meta-analysis (Supplementary Results, Table S8, and Figure S2).

**Table 2:** Differences in cortical thickness between medication-free patients with major depressive disorder and healthy controls

Region	MNI coordinate			SDM *Z* score	*P*, uncorrected	Voxels	Cluster breakdown (voxels)
	*x*	*y*	*z*				
*Patients with major depressive disorder* *>* *healthy controls*							
Left posterior cingulate cortex	0	−24	34	1.113	0.00135	471	Left posterior cingulate/paracingulate gyri, BA 23 (206)
							Right posterior cingulate/paracingulate gyri, BA 23 (141)
							Median network, cingulum (124)
Right ventromedial prefrontal cortex	8	50	2	1.178	0.00087	208	Right superior frontal gyrus, medial, BA 10 (91)
							Left anterior cingulate/paracingulate gyri, BA 24, 32 (85)
							Left median network, cingulum (23)
							Right anterior cingulate/paracingulate gyri, BA 10, 24, 32 (9)
*Patients with major depressive disorder* *<* *healthy controls*							
Left gyrus rectus	−4	32	−26	−1.412	0.00027	629	Left gyrus rectus, BA 11 (491)
							Left superior frontal gyrus, orbital part, BA 11 (78)
							Left superior frontal gyrus, medial orbital, BA 11 (47)
							Right gyrus rectus, BA 11 (13)
Right middle temporal gyrus	46	−72	10	−1.144	0.00146	89	Right middle temporal gyrus, BA 19, 37, 39 (78)
							Right middle occipital gyrus, BA 19, 37 (11)
Left superior frontal gyrus, orbital part	−16	58	−8	−1.162	0.00132	63	Left superior frontal gyrus, orbital part, BA 11 (60)
							Left middle frontal gyrus, orbital part, BA 11 (3)

*BA* Brodmann area, *MNI* Montreal Neurological Institute, *SDM* seed-based *d* mapping

### Jackknife, heterogeneity and publication bias analysis

In whole-brain jackknife sensitivity analysis, decreased cortical thickness in left gyrus rectus, left oSFG and right MTG, and increased cortical thickness in right vmPFC extending to left ACC, were preserved in 14 combinations of 15 datasets, while increased cortical thickness in the left PCC remained statistically significant in 13/15 datasets (Table S5); the results of the pooled meta-analysis thus showed high replicability and reliability in those regions. So did the jackknife sensitivity analysis of the subgroup meta-analyses in studies with non-comorbid patients and threshold correction (Tables S6 and S7). For the jackknife sensitivity analysis of the subgroup meta-analysis in studies with medication-naive patients with MDD, the cortical thickness in the orbital part of right middle frontal gyrus and left oSFG were preserved in six combinations of seven studies while the finding in right vmPFC was preserved in four combinations (Table S9).

**Fig. 3:**
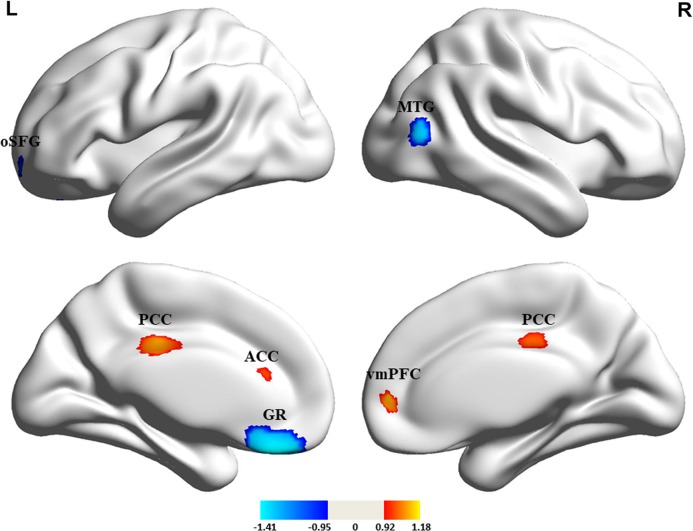
Cortical thickness alterations in medication-free patients with major depressive disorder compared with healthy controls. Regions of increased (warm color) and decreased (cool color) cortical thickness in medication-free patients with MDD than HCs in the pooled meta-analysis. ACC, anterior cingulate cortex; GR, gyrus rectus; L, left; oSFG, orbital segment of the superior frontal gyrus; PCC, posterior cingulate cortex; vmPFC, ventromedial prefrontal cortex; MTG; middle temporal gyrus; R, right

In the pooled meta-analysis, none of the regions with altered cortical thickness (Table 2 and Fig. 3) showed statistically significant heterogeneity between studies. In the analysis of publication bias, the Egger test of funnel plot asymmetry was not statistically significant in the left PCC (*Z* = 1.21, *t* = 1.00, *df* = 13, *P* = 0.335), right vmPFC extending to left ACC (*Z* = −0.27, *t* = −0.21, *df* = 13, *P* = 0.834), left gyrus rectus (*Z* = −1.41, *t* = −0.83, *df* = 13, *P* = 0.419), left oSFG (*Z* = 0.95, *t* = 0.90, *df* = 13, *P* = 0.440), or right MTG (*Z* = 0.02, *t* = 0.01, *df* = 13, *P* = 0.989) (Figure S1).

### Meta-regression analysis

**Fig. 4:**
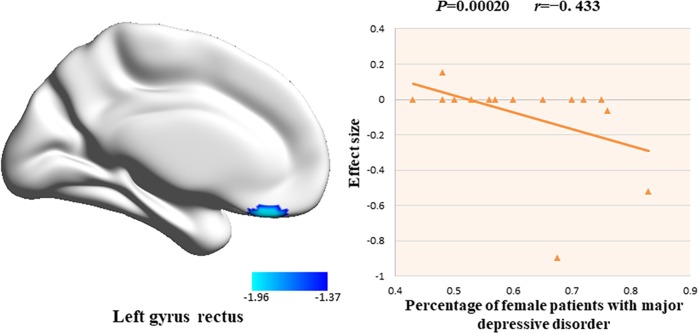
The results of meta-regression analysis. The percentage of female patients with the major depressive disorder was negatively correlated with cortical thickness in the left gyrus rectus in the meta-regression. The effect sizes to create this plot were extracted from the peak of the maximum slope difference and each study is represented as a dot. The regression line (meta-regression signed differential mapping slope) is shown

The percentage of female patients with MDD was negatively associated with decreased cortical thickness in the left gyrus rectus (*r* = −0.433, *P* = 0.00020) (Fig. 4). This result was driven by 4 studies [12, 15, 17, 29]. Neither mean age of patients, age at onset, illness duration, number of episodes, or illness severity were statistically significantly associated with cortical thickness alterations.

## Discussion

To our knowledge, this is the first meta-analysis of vertex-based FreeSurfer studies to identify a significant and complex pattern of cortical thickness alteration in untreated patients with MDD. It also incorporates a significant methodological innovation. A new mask was specifically designed for the meta-analysis of cortical thickness and will be made freely available in SDM software for use in future meta-analyses. Using the new mask, we identified replicable increased cortical thickness in the default mode network (DMN) (PCC, vmPFC, and ACC), and decreased cortical thickness in OFC (gyrus rectus and oSFG) and temporal cortex in medication-free patients with MDD. In addition, we found a negative correlation between the percentage of female patients and cortical thickness alterations in gyrus rectus.

Previous meta-analyses reported gray-matter loss (decreased gray-matter volume/density) in the frontal cortex, temporal lobe, OFC and cingulate cortex in patients with MDD [31, 32], regions with observed alterations in our study. Consistent with the previous meta-analysis conducted by the ENIGMA consortium [20], we identified cortical thickness alterations in OFC, ACC, PCC, and MTG. Of note, we found increased cortical thickness in ACC and PCC, while the ENIGMA consortium reported changes in the opposite direction. The difference in findings might be related to the ENIGMA patients being typically treated and later in illness course than participants of studies in our meta-analysis. Compared with the study by Suh et al. [21], we did not observe cortical thickness abnormalities in pars opercularis, calcarine fissure/lingual gyrus or supramarginal gyrus in patients with MDD. The reasons for these differences might be attributable to the heterogeneities of mask selection and medication status of patients with MDD.

### Increased cortical thickness in the DMN in MDD

Understanding of the pathophysiology of MDD has progressed steadily, including improved understanding of the role of genes, inflammation, and changes in brain anatomy and function [3]. Previous neuroimaging studies in MDD found structural and functional brain abnormalities mainly in the medial prefrontal-limbic circuit (areas modulated by serotonin neurotransmission and related to emotion regulation) and the orbitofrontal-striatal network (modulated by dopamine and underpinning reward processing) [33, 34]. Previous studies focused on the connectivity stability in MDD have also demonstrated the great importance of DMN [35, 36]. Patients with MDD show a stronger functional correlation between the anterior parts of DMN and the amygdala during self-referential processing and elevated functional connectivity (FC) between the dorsal and rostral parts of ACC during cognitive and affective challenges [37]. A meta-analysis of resting-state FC in MDD has confirmed functional hyper-connectivity within DMN [38]. Patients with MDD also show greater DMN activity in the resting-state and less deactivation of that network during cognitive processing [39]. The brain regions with increased cortical thickness identified in the present meta-analysis (PCC, vmPFC, and ACC) are important hubs in the DMN [40], which is involved in affective processing and self-referential processing [41]. These structural alterations may be related to the failures to deactivate DMN regions during cognitive activity, and perhaps represent a consequence of neuronal hyperactivity over time in the regions.

Yan et al. found decreased DMN FC in recurrent MDD (mean illness duration 7.7 years), which was associated with medication usage [42], while Kim and colleagues observed increased DMN FC in the first-episode, drug-naive patients with MDD [43]. These divergent findings suggest a potential trajectory of FC in DMN over the course of the illness in MDD. In our study, the median illness duration was only 1.2 years in 11 of our studies, so that our findings of increased thickness in DMN regions may align with increased functional connectivity reported in the Kim study.

The vmPFC and ACC are involved with mood regulation and higher-order cognitive processing [44, 45], and increased cortical thickness in these areas might be related to symptoms including anhedonia, negative thinking, and changes in emotional experience associated with MDD [45]. Functional imaging evidence has reported ACC hyperactivation in MDD, which was a predictive biomarker for treatment response [46]. The left PCC, another region with increased cortical thickness, plays an essential role in arousal, and internal vs. external focus of thought and attentional focus [47]. The vmPFC can regulate PCC activity that is enhanced during explicit self-appraisal; in MDD, and alteration of this regulation may contribute to disturbances of self-appraisal that are a critical feature of the illness [45]. van Eijndhoven et al. found that increased cortical thickness in caudal ACC and PCC were trait-related in first-episode patients with MDD [12], suggesting that ACC and PCC alterations may play a vital role in the early stage of MDD.

The mechanism of increased cortical thickness in MDD is unclear, although one could speculate that it may result from the dual activation of the immune-inflammatory response system and the compensatory immune-regulatory reflex system [48]. Possible causes of increased cortical thickness observed in some brain regions include pre-apoptotic osmotic changes (partly induced by increased cytokines), cellular hypertrophy, and cytokine-activated astrocyte proliferation [49, 50]. For instance, interleukin-1β, which has been reported to be increased in patients with MDD [51], could induce astrocyte nuclear hypertrophy [52]. Astrocytes play a critical role in the blood-brain barrier; their energy metabolism helps prevent excitotoxicity and they release neurotrophic factors which assist neuronal survival and formation of new synapses [49]. Such compensatory mechanisms may be particularly relevant early in MDD, as cortical thickness and activity seem to be increased in some regions early in the illness course but decrease as the illness progresses. While we did not observe any statistically significant correlation between cortical thickness and illness duration in our study of untreated patients, this may be due to the typically short illness duration of samples recruited in studies included in our meta-analysis. The exact pathological mechanisms underlying increased cortical thickness in medication-free patients with MDD requires further investigation.

### Orbitofrontal and temporal cortical thinning in MDD

Decreased cortical thickness was seen in OFC regions including the left gyrus rectus and the oSFG. The OFC contributes to reward processing, exteroceptive and interoceptive information coding, impulse control, mood regulation, and decision making [53], and so OFC abnormalities may contribute to commonly observed emotional and cognitive impairments in depression. Consistent with our finding of decreased cortical thickness in these regions, both structural (decreased gray-matter volumes [54]) and functional (decreased regional homogeneity [55], cerebral blood flow [56], and amplitude of low-frequency fluctuations [56]) studies in MDD have reported abnormalities in OFC. In addition, van Eijndhoven et al. reported that decreased thickness of OFC was related to trait anxiety and altered mood regulation in MDD [12]. Peterson et al. found decreased cortical thickness in left OFC in individuals at increased familial risk for MDD [57], suggesting that cortical thinning of left OFC may precede illness onset or reflect alterations related to risk for MDD.

A higher percentage of female patients with MDD in studies included in current meta-analysis was associated with the decreased cortical thickness in the left gyrus rectus. Sex difference in MDD is important, as females show a prevalence of MDD in nearly twice that of males [58], have a higher rate of psychiatric comorbidities and make more frequent suicide attempts [59]. A previous meta-analysis of gray-matter volume studies of MDD found smaller prefrontal cortex in female than male patients [54], consistent with our findings.

Interestingly, OFC expresses high concentrations of glucocorticoid receptors and thus is vulnerable to injury induced by increased glucocorticoid levels [60], including increased sensitivity to excitotoxic injury and neuron death [61]. Consistent with this, a negative correlation has been reported between cortical thickness in OFC and serum cortisol levels [28]. There is also evidence that increased interleukin-6 is inversely correlated with the cortical thickness of OFC in MDD [13], and that it controls the expression of the serotonin transporter and consequently serotonin reuptake, with potential relevance for behavioral features of MDD [62]. In addition, high-sensitivity C-reactive protein has been related to reduced gray-matter volume in the prefrontal cortex, indicating the potential role of inflammatory activation in the anatomic alterations associated with MDD [63]. Further, there is postmortem evidence for a reduced density of pyramidal neurons in OFC that could contribute to the decreased cortical thickness of OFC observed in our meta-analysis of MDD [64].

The area of posterior MTG identified to show reduced cortical thickness is well known as a region controlling the perception of dynamic moving objects [65, 66]. This region is important for regulating sensorimotor responses to visual motion, which are impaired in MDD [67]. Alterations in this region may contribute to the perceptual analysis of social and other dynamic visual events. In addition, the MTG plays an important role in facial emotion perception, which is related to nonverbal social communication, and thus MTG abnormalities might be expected to affect interpersonal engagement and social functioning [68, 69]. Previous neuroimaging studies of MDD have reported decreased gray-matter volume [70], reduced functional activity [71], and disrupted functional network connectivity in MTG [72]. Our results, taken together with these, are consistent with a role for MTG alterations in MDD.

### Potential etiological implications

One of the most striking features of our meta-analysis was the demonstration of a complex pattern of regional dystrophic and hypertrophic alterations in MDD evident across studies of medication-free patients with MDD. While our meta-analysis summarizes this pattern of effects, it also raises questions and suggests paths for productive future research. Previous evidence shows that the alterations in immune-inflammatory systems can be associated with both increased and decreased cortical thickness. Poletti et al. observed that increased inflammatory markers such as tumor necrosis factor-α, interleukin-8, and chemokine (CCmotif) ligand were correlated with the increased cortical thickness of ACC in patients with bipolar depression [73]. Piras and colleagues observed that increased transforming growth factor β (TGF-β) was associated with increased cortical thickness in cingulate and frontal areas in healthy individuals [74]. The role of TGF-β in downregulating inflammatory processes and in promoting repair mechanisms might be related to the observed increased cortical thickness [75]. One study of mice showed that maternal immune activation during pregnancy was associated with increased cortical thickness in their offspring [76]. Kakeda et al. found that reduced cortical thickness in orbital frontal cortex in patients with MDD was negatively correlated with increased interleukin-6 levels [13].

Based on the evidence that the OFC expresses high concentrations of glucocorticoid receptors mentioned above [77], while Alt et al. reported the MDD patients exhibited significantly decreased glucocorticoid receptor-α density in the cingulate cortex [78], such factors might account for variable patterns of cortical thickness changes across the brain rather than a consistent effect across cortex. We speculated that cortical thickening in certain brains regions and cortical thinning in other brain regions might be due to heterogeneities of response sensitivity or differing time course of evolving neuronal alterations related to illness pathophysiology in MDD. Why immune-inflammatory related factors would alter regional brain activity and anatomy selectively in this way remains to be determined. Further neurobiological and neurobehavioral studies of these two patterns of alterations are needed to clarify their clinical significance and underlying mechanisms.

### Limitations

First, like most coordinate-based meta-analysis studies, we summarized data (i.e. reported coordinates) rather than raw data from individual cases, which limits precision in the characterization of the precise and full spatial location of effects. Furthermore, we could not obtain coordinates for all prior studies, despite our efforts to contact the authors of otherwise suitable studies [79–83]. Second, our meta-regression finding that the percentage of female patients was negatively correlated with cortical thickness in left gyrus rectus was driven by four studies, and thus it requires further confirmation. Third, since the present meta-analysis focused on cross-sectional studies, longitudinal studies are needed to determine the degree to which cortical thickness changes in MDD diminish or progress after clinical recovery and antidepressant therapy. Fourth, because the number and sample sizes of prior studies of drug-naive patients were limited, we included studies of previously treated patients who were untreated before MRI scanning. Although previous study reported no statistically significant differences in resting-state brain activity between drug-naive and currently off-medication patients with MDD [84], the potential influences of prior medication on cortical thickness could not be completely excluded. Clearly, the best way to minimize the effects of medication on brain measures is to focus on drug-naive patients with MDD because the drug-naive and untreated conditions may not be the same in relation to associated brain abnormalities. However, studying currently untreated patients is of interest to determine whether different alterations are present from those associated with the never treated illness that may indicate “scars” of prior episodes or enduring effects of prior treatment. Meta-analysis comparing medication-naive and currently off-medication patients with MDD are needed when the number of published studies of both types permits. Finally, there were some potential limitations of this specific meta-analytic mask. An obvious limitation (and benefit) of the new mask is that it only includes cortical gray matter. Therefore, we recommend the standard gray-matter mask (instead of the new mask) for meta-analyses that include subcortical gray matter (e.g. VBM). Another limitation related to the mask is that we created the mask based on the FreeSurfer mask, and this may have slight differences with other surface-based analysis software and procedures. We suspect that such differences will be minor and thus have a small or negligible impact on results.

## Conclusion

In conclusion, by applying a new mask in SDM and focusing on the medication-free patient with MDD for the present meta-analysis, we identified significant regional cortical thickness alterations in MDD, including increased cortical thickness in DMN (PCC, vmPFG, and ACC) and decreased cortical thickness in OFC and MTG. Meta-regression suggests that female medication-free patients with MDD may have a lower cortical thickness in left gyrus rectus. These findings in untreated patients where pharmacological treatment effects could have a limited impact on brain measurements identified important new information about intrinsic structural brain alterations in patients with MDD.

## Funding and disclosure

This study was supported by the National Natural Science Foundation (Grant Nos. 81621003, 81761128023, 81820108018, 81227002, and 81030027), National Key Technologies R&D Program (Program No. 2012BAI01B03) and Program for Changjiang Scholars and Innovative Research Team in University (PCSIRT, Grant No. IRT16R52) of China. Dr. Gong would also like to acknowledge the support from his Changjiang Scholar Professorship Award (Award No. T2014190) of China and American CMB Distinguished Professorship Award (Award No. F510000/G16916411) administered by the Institute of International Education, USA. Dr. Li would like to acknowledge the support from the Sichuan Science and Technology Program (2019YJ0098), Science and Technology Project of the Health Planning Committee of Sichuan (18ZD035 and 19PJ078), Technology Foundation for the Selected Returned Overseas Chinese Scholars (Sichuan Provincial Human Resources and Social Security Department, [2018]145-19), and Fundamental Research Funds for the Central Universities (2018SCUH0011). All authors declare no biomedical financial interests or potential conflicts of interest.

## Supplementary information

Supplementary Materials
