# Correction: Meta-analysis of cortical thickness abnormalities in medication-free patients with major depressive disorder

**DOI:** 10.1038/s41386-019-0587-1

**Published:** 2019-12-12

**Authors:** Qian Li, Youjin Zhao, Ziqi Chen, Jingyi Long, Jing Dai, Xiaoqi Huang, Su Lui, Joaquim Radua, Eduard Vieta, Graham J. Kemp, John A. Sweeney, Fei Li, Qiyong Gong

**Affiliations:** 1 Huaxi MR Research Center (HMRRC), Functional and Molecular Imaging Key Laboratory of Sichuan Province, Department of Radiology, West China Hospital of Sichuan University, Chengdu, Sichuan 610041 P. R. China; 2 Psychoradiology Research Unit of Chinese Academy of Medical Sciences (2018RU011), West China Hospital of Sichuan University, Chengdu, 610041 China; 3 Department of Psychoradiology, The Fourth People’s Hospital of Chengdu, Chengdu, China; 4 Institut d’Investigacions Biomèdiques August Pi i Sunyer (IDIBAPS), Mental Health Research Networking Center (CIBERSAM), Barcelona, Spain; 5 Centre for Psychiatric Research and Education, Department of Clinical Neuroscience, Karolinska Institutet, Stockholm, Sweden; 6 Department of Psychosis Studies, Institute of Psychiatry, Psychology and Neuroscience, King’s College London, London, UK; 7 Barcelona Bipolar Disorders and Depressive Unit, Hospital Clinic, Institute of Neurosciences, University of Barcelona, Barcelona, Spain; 8 Liverpool Magnetic Resonance Imaging Centre (LiMRIC) and Institute of Ageing and Chronic Disease, University of Liverpool, Liverpool, UK; 9 Department of Psychiatry, University of Cincinnati, Cincinnati, OH USA

**Keywords:** Diagnostic markers, Depression

**Correction to:** *Neuropsychopharmacology* 10.1038/s41386-019-0563-9, published online 6 November 2019

The original version of this Article contained an error in the author affiliations.

The affiliation of Su Lui with Huaxi MR Research Center (HMRRC), Functional and Molecular Imaging Key Laboratory of Sichuan Province, Department of Radiology, West China Hospital of Sichuan University, Chengdu, Sichuan 610041, P. R. China was inadvertently omitted.

This has now been corrected in both the PDF and HTML versions of the Article.

