# Supplementary material for: Meta-analysis of cortical thickness abnormalities in medication-free patients with major depressive disorder
Source: Neuropsychopharmacology. 2019 Nov 6;45(4):703–12. doi: 10.1038/s41386-019-0563-9 (PMC7021694; doi:10.1038/s41386-019-0563-9)
Supplement: Supplementary file 1 — Supplementary Materials [file 41386_2019_563_MOESM1_ESM.doc]

**Supplementary Materials**

**Content of supplementary materials**

Supplementary Methods

Supplementary Results

Table S1. PRISMA 2009 Checklist.

Table S2. The checklist of imaging methodology quality assessment for all the articles included in the present meta-analysis.

Table S3. Differences in cortical thickness between non-comorbid patients with MDD and HCs.

Table S4. Differences in cortical thickness between the medication-free patients with MDD and HCs in studies with threshold correction.

Table S5. Results of the jackknife analysis in all included studies.

Table S6. Results of the jackknife analysis in studies with non-comorbid patients.

Table S7. Results of the jackknife analysis in studies with threshold correction.

Table S8. Differences in cortical thickness between medication-naive patients with MDD and HCs.

Table S9. Results of the jackknife analysis in studies with medication-naive patients.

Figure S1. Results of funnel plot for the publication bias analysis.

Figure S2. Cortical thickness alterations in medication-naive patients with MDD.

**Supplementary Methods**

## 1. Search strategy and selection criteria

The current meta-analysis was performed according to the Preferred Reporting Items for Systematic reviews and Meta-Analyses guidelines (PRISMA) (Table S1) [1]. A comprehensive and systematic literature search was conducted in PubMed, Web of Science, Embase, and Science Direct up to July 14, 2018, using the keywords “depression” or “depressive disorder” or “unipolar depression” plus “cortical thickness” or “thickness”. Additional publications were identified by manual searches in reference lists. Original structural MRI studies were included according to these criteria: 1) used a vertex-based method to estimate whole-brain cortical thickness changes in MDD; 2) compared patients with MDD to HCs; 3) examined adults, excluding individuals >65 years [2] or <18 years old [3]; 4) were limited to medication-free patients, who either had never received anti-depressant medication (i.e. were medication-naive) or who were previously treated but drug free for at least 7 days prior to MRI scanning; and 5) provided the effect coordinates of significant clusters in Montreal Neurological Institute (MNI) or Talairach space. Eligible studies reporting no between-group differences were included and estimated conservatively to have a null effect size in SDM. Theoretical papers, reviews and case reports were excluded, as well as studies limited to regions-of-interest (ROIs) analysis and studies focused on peri- or postmenopausal female participants. The studies that did not perform statistical comparisons between patients and controls and for which peak coordinates of effects were not available even after contacting with the authors were excluded in the current meta-analysis. Two of us (Q.L. and Y.J.Z.) conducted the literature search, independently. The results of these two searches were compared, and any inconsistencies were discussed and a consensus decision was reached about the appropriateness of the study for this meta-analysis.

## 2. Quality assessment and data recording

We used a 12-point checklist to estmate the quality of each included study, based on the reported demographic and clinical characteristics of the participants and the imaging methodology [4]. Each point was scored as 0, 0.5 or 1 if the criteria were unfulfilled, partially met or fully met, respectively, and any study scoring >6.0 was included in the meta-analysis. Although not specifically designed as an assessment tool, this checklist provided an objective indication of the rigor of each study to warrant inclusion in this analysis (Table S2). Data recorded from the review of each included article were of two kinds. First, we recorded demographic and clinical characteristics (sample size, gender, mean age, age at onset, mean number of episodes, illness duration, depression symptom severity, and medication status) and basic methodological information (statistical threshold of main findings and the method used to correct whole-brain results for multiple comparisons) as well as scanning parameters (magnetic field strength and voxel size). Second, we recorded the data for SDM calculations, including the coordinates of main findings and values related to effect size (e.g. *t* statistics, *Z* score, and *P* value) [5].

## 3. Meta-analysis of abnormalities in cortical thickness

Details of the SDM method have been published previously [6,7], and we thus provide only a brief summary. First, SDM used the coordinates of cluster peaks and the effect sizes of significant differences between patients and HCs to create an effect-size signed map for each study by means of an anisotropic Gaussian kernel. When selecting coordinates the same threshold was applied throughout the whole brain in each study, to avoid bias towards regions with liberal thresholds (e.g. prior ROIs), although it is not necessary to demand the same threshold in every study. Then we used the default 20 mm full-width at half-maximum to assign indicators of proximity to reported coordinates, but not to smooth any image that is different in nature [6], and chose the new mask to restrict the analysis to the cortical regions. Next, SDM performed random-effects analysis to obtain the mean map, combining the data of each included study and representing both negative and positive differences in the same map [7]. Finally, SDM carried out sensitivity and heterogeneity analyses to evaluate the meta-analytic outcome. We used SDM’s default thresholds (voxel threshold *P*<0.005 with peak *Z*>1 and a cluster extent of 50 voxels) and show results in MNI coordinates. Taking into account the heterogeneities of clinical characteristics and methodology, we also performed the subgroup meta-analysis if the number of studies was sufficient.

## 4. Jackknife, heterogeneity and publication bias analysis

To test the replicability of the results, we conducted a systematic whole-brain jackknife sensitivity analysis in the pooled and subgroup meta-analyses, by repeating the main analysis *n* times (*n*=the number of datasets included), discarding one study at a time to determine whether the results remained detectable. We estimated the statistical (between-studies) heterogeneity of individual clusters using *Q* statistics (*χ2* distribution converted to *z* values) and tested for increased heterogeneity of findings with a permutation approach, in which any result presenting voxel threshold *P*<0.005 with peak *Z*>1 and cluster extent of 50 voxels was considered to have significantly elevated heterogeneity. Publication bias was examined with Egger tests to assess the asymmetry of funnel plots for each significant cluster of patient-control comparisons, in which any result showing *P*<0.05 was significant for publication bias [8].

## 5. Meta-regression analysis

The variables explored in meta-regression analysis were: mean age of patients, the percentage of female patients, age at onset, illness duration, the mean number of episodes, and severity of illness. The probability threshold was decreased to 0.0005 to minimize detection of spurious relationships [7]. We required that findings be detected both in one of the extremes of the regressor in the slope and, and discarded any results not significant in the main meta-analysis [7,9]. Finally, the regression plots were visually inspected to discard findings driven by too few studies [7].

**Supplementary Results**

In order to evaluate potential medication effects, we conducted a subgroup meta-analysis of medication-naive patients involving 7 of the original studies [10-16]. The results showed increased cortical thickness in the orbital segment of right middle frontal gyrus (*Z*=1.158, *P*=0.00230) and right ventromedial prefrontal cortex (*Z*=1.156, *P*=0.00235), as well as decreased cortical thickness in the orbital segment of left superior frontal gyrus (*Z*=−1.306, *P*=0.00132) (Table S8 and Figure S2).

**Table S1. PRISMA 2009 Checklist*.**

| **Section/topic** | **#** | **Checklist item** | **Reported on page #** |
| --- | --- | --- | --- |
| **TITLE** | | |  |
| Title | 1 | Identify the report as a systematic review, meta-analysis, or both. | 1 |
| **ABSTRACT** | | |  |
| Structured summary | 2 | Provide a structured summary including, as applicable: background; objectives; data sources; study eligibility criteria, participants, and interventions; study appraisal and synthesis methods; results; limitations; conclusions and implications of key findings; systematic review registration number. | 3 |
| **INTRODUCTION** | | |  |
| Rationale | 3 | Describe the rationale for the review in the context of what is already known. | 4-6 |
| Objectives | 4 | Provide an explicit statement of questions being addressed with reference to participants, interventions, comparisons, outcomes, and study design (PICOS). | 6-7 |
| **METHODS** | | |  |
| Protocol and registration | 5 | Indicate if a review protocol exists, if and where it can be accessed (e.g., Web address), and, if available, provide registration information including registration number. | 8, s2 |
| Eligibility criteria | 6 | Specify study characteristics (e.g., PICOS, length of follow-up) and report characteristics (e.g., years considered, language, publication status) used as criteria for eligibility, giving rationale. | s2 |
| Information sources | 7 | Describe all information sources (e.g., databases with dates of coverage, contact with study authors to identify additional studies) in the search and date last searched. | s2 |
| Search | 8 | Present full electronic search strategy for at least one database, including any limits used, such that it could be repeated. | s2 |
| Study selection | 9 | State the process for selecting studies (i.e., screening, eligibility, included in systematic review, and, if applicable, included in the meta-analysis). | s2 |
| Data collection process | 10 | Describe method of data extraction from reports (e.g., piloted forms, independently, in duplicate) and any processes for obtaining and confirming data from investigators. | s2 |
| Data items | 11 | List and define all variables for which data were sought (e.g., PICOS, funding sources) and any assumptions and simplifications made. | s3 |
| Risk of bias in individual studies | 12 | Describe methods used for assessing risk of bias of individual studies (including specification of whether this was done at the study or outcome level), and how this information is to be used in any data synthesis. | s3-5 |
| Summary measures | 13 | State the principal summary measures (e.g., risk ratio, difference in means). | 8-9, s3-5 |
| Synthesis of results | 14 | Describe the methods of handling data and combining results of studies, if done, including measures of consistency (e.g., I^2^) for each meta-analysis. | 8-9, s3-5 |
| Risk of bias across studies | 15 | Specify any assessment of risk of bias that may affect the cumulative evidence (e.g., publication bias, selective reporting within studies). | s3-5 |
| Additional analyses | 16 | Describe methods of additional analyses (e.g., sensitivity or subgroup analyses, meta-regression), if done, indicating which were pre-specified. | s3-5 |
| **RESULTS** | | |  |
| Study selection | 17 | Give numbers of studies screened, assessed for eligibility, and included in the review, with reasons for exclusions at each stage, ideally with a flow diagram. | 10-12 |
| Study characteristics | 18 | For each study, present characteristics for which data were extracted (e.g., study size, PICOS, follow-up period) and provide the citations. | 10-11 |
| Risk of bias within studies | 19 | Present data on risk of bias of each study and, if available, any outcome level assessment (see item 12). | 13 |
| Results of individual studies | 20 | For all outcomes considered (benefits or harms), present, for each study: (a) simple summary data for each intervention group (b) effect estimates and confidence intervals, ideally with a forest plot. | 12 |
| Synthesis of results | 21 | Present results of each meta-analysis done, including confidence intervals and measures of consistency. | 12-13 |
| Risk of bias across studies | 22 | Present results of any assessment of risk of bias across studies (see Item 15). | 13 |
| Additional analysis | 23 | Give results of additional analyses, if done (e.g., sensitivity or subgroup analyses, meta-regression [see Item 16]). | 14 |
| **DISCUSSION** | | |  |
| Summary of evidence | 24 | Summarize the main findings including the strength of evidence for each main outcome; consider their relevance to key groups (e.g., healthcare providers, users, and policy makers). | 14-22 |
| Limitations | 25 | Discuss limitations at study and outcome level (e.g., risk of bias), and at review-level (e.g., incomplete retrieval of identified research, reporting bias). | 22-23 |
| Conclusions | 26 | Provide a general interpretation of the results in the context of other evidence, and implications for future research. | 24 |
| **FUNDING** | | |  |
| Funding | 27 | Describe sources of funding for the systematic review and other support (e.g., supply of data); role of funders for the systematic review. | 25-26 |

*Note: Moher D, Liberati A, Tetzlaff J, Altman DG, The PRISMA Group (2009). Preferred Reporting Items for Systematic Reviews and Meta-Analyses: The PRISMA Statement. PLoS Med 6(7): e1000097. doi:10.1371/journal.pmed1000097. For more information, visit www.prisma-statement.org.

Abbreviations: s, supplementary materials

**Table S2. The checklist of imaging methodology quality assessment for all the articles included in the present meta-analysis.**

| **12-point checklist** | Han[10] | Kakeda[11] | Lan[17] | Liu[12] | Na[13] | Niu[18] | Peng[14] | Qiu[15] | Späti[19] | Taylor[20] | Van[21] | Van[22] | Wagner[23] | Yang[16] | Zorlu[24] |
| --- | --- | --- | --- | --- | --- | --- | --- | --- | --- | --- | --- | --- | --- | --- | --- |
| **Category 1: Subjects** |  |  |  |  |  |  |  |  |  |  |  |  |  |  |  |
| 1. Patients were evaluated prospectively, specific diagnostic criteria were applied, and demographic data were reported | 1 | 1 | 1 | 1 | 1 | 1 | 1 | 1 | 1 | 1 | 1 | 1 | 1 | 1 | 1 |
| 2. Healthy comparison participants were evaluated prospectively; psychiatric and medical illnesses were excluded | 1 | 1 | 1 | 1 | 1 | 1 | 1 | 1 | 1 | 1 | 1 | 1 | 1 | 1 | 1 |
| 3. Important variables (e.g., age, gender, drug status, illness duration, and symptom severity) were checked either via stratification or statistics | 0.5 | 0.5 | 0.5 | 0.5 | 0.5 | 1 | 1 | 0.5 | 0.5 | 0.5 | 1 | 0.5 | 1 | 0.5 | 0.5 |
| 4. All patients were comorbidity free | 1 | 1 | 1 | 1 | 1 | 1 | 1 | 1 | 1 | 1 | 1 | 1 | 1 | 1 | 1 |
| 5. All patients were medication naive | 1 | 1 | 0.5 | 1 | 1 | 0.5 | 1 | 1 | 0.5 | 0.5 | 0.5 | 0.5 | 0.5 | 1 | 0.5 |
| 6. Sample size per group: ≥ 20, scores 1; ≥ 10, scores 0.5 | 1 | 1 | 1 | 1 | 1 | 1 | 0.5 | 1 | 1 | 1 | 1 | 1 | 1 | 1 | 1 |
| **Category 2: Methods for image acquisition and analysis** | | | | | | | | | | | | | | | |
| 7. Magnet strength: 3T, scores 1; 1.5T, scores 0.5 | 1 | 1 | 1 | 1 | 1 | 1 | 1 | 1 | 1 | 1 | 0.5 | 0.5 | 0.5 | 0.5 | 0.5 |
| 8. The imaging technique used was clearly described so that it could be reproduced | 1 | 1 | 1 | 1 | 1 | 1 | 1 | 1 | 1 | 1 | 1 | 1 | 1 | 1 | 1 |
| 9. Whole brain analysis was automated without a previously defined region | 1 | 1 | 1 | 1 | 1 | 1 | 1 | 1 | 1 | 1 | 1 | 1 | 1 | 1 | 1 |
| 10. Spatial coordinates were reported in a standard space (e.g., Talairach or MNI coordinates) | 1 | 1 | 1 | 1 | 1 | 1 | 1 | 1 | 1 | 1 | 0.5 | 1 | 1 | 0.5 | 1 |
| **Category 3: Results and conclusions** |  |  |  |  |  |  |  |  |  |  |  |  |  |  |  |
| 11. Statistical results were corrected for multiple comparison scores 1, uncorrected scores 0.5 | 1 | 1 | 1 | 1 | 1 | 1 | 1 | 0.5 | 1 | 1 | 1 | 1 | 1 | 1 | 1 |
| 12. Conclusions were consistent with the results obtained, and the limitations were discussed | 1 | 1 | 1 | 1 | 1 | 1 | 1 | 1 | 1 | 1 | 1 | 1 | 1 | 1 | 1 |
| **Total score** | 11.5 | 11.5 | 11 | 11.5 | 11.5 | 11.5 | 11.5 | 11 | 11 | 11 | 10.5 | 10.5 | 11 | 10.5 | 10.5 |

**Table S3. Differences in cortical thickness between non-comorbid patients with MDD and HCs (12 studies [10-15,18,20-24]).**

| **Region** | **MNI coordinate** | | | **SDM** | ***P***, uncorrected | | **Voxels** | **Cluster breakdown** (voxels) |
| --- | --- | --- | --- | --- | --- | --- | --- | --- |
|  | x | y | z | ***Z* score** |  |  |  |  |
| **Non-comorbid patients with major depression disorder > healthy controls** | | | | | | | | |
| Left posterior cingulate cortex | 0 | -14 | 34 | 1.285 | 0.00134 | | 979 | Left posterior cingulate / paracingulate gyri, BA 23 (389) |
|  |  |  |  |  |  |  |  | Right posterior cingulate / paracingulate gyri, BA 23 (320) |
|  |  |  |  |  |  |  |  | Median network, cingulum (270) |
| Right ventromedial prefrontal cortex | 6 | 50 | 2 | 1.332 | 0.00091 | | 283 | Right superior frontal gyrus, medial, BA 10 (120) |
|  |  |  |  |  |  |  |  | Left anterior cingulate / paracingulate gyri, BA 24, 32 (147) |
|  |  |  |  |  |  |  |  | Left median network, cingulum (16) |
| **Non-comorbid patients with major depression disorder < healthy controls** | | | | | | | | |
| Left gyrus rectus | -4 | 32 | -26 | -1.438 | 0.00040 | | 1023 | Left gyrus rectus, BA 11 (493) |
|  |  |  |  |  |  |  |  | Left superior frontal gyrus, medial/orbital part, BA 11 (233) |
|  |  |  |  |  |  |  |  | Right gyrus rectus, BA 11 (145) |
|  |  |  |  |  |  |  |  | Right superior frontal gyrus, medial/orbital, BA 11 (88) |
|  |  |  |  |  |  |  |  | Olfactory cortex, BA 11, 25 (64) |
| Right middle temporal gyrus | 44 | -72 | 12 | -1.204 | | 0.00167 | 109 | Right middle temporal gyrus, BA 19, 37, 39 (95) |
|  |  |  |  |  |  |  |  | Right middle occipital gyrus, BA 19, 37 (14) |
| Left superior frontal gyrus, orbital part | -14 | 58 | -8 | -1.215 | | 0.00153 | 75 | Left superior frontal gyrus, orbital part, BA 11 (71) |
|  |  |  |  |  |  |  |  | Left middle frontal gyrus, orbital part, BA 11 (4) |
| Abbreviations: BA, Brodmann area; MNI, Montreal Neurological Institute; SDM, Seed-based d Mapping. | | | | | | | | |

**Table S4. Differences in cortical thickness between the medication-free patients with MDD and HCs in studies with threshold correction (14 studies [10-15,17-24]).**

| **Region** | **MNI coordinate** | | | **SDM** | ***P***, uncorrected | **Voxels** | **Cluster breakdown** (voxels) |
| --- | --- | --- | --- | --- | --- | --- | --- |
|  | x | y | z | ***Z* score** |  |  |  |
| **Patients with major depression disorder > healthy controls** | | | | | | | |
| Left posterior cingulate cortex | 0 | -18 | 34 | 1.238 | 0.00121 | 962 | Left posterior cingulate / paracingulate gyri, BA 23 (400) |
|  |  |  |  |  |  |  | Right posterior cingulate / paracingulate gyri, BA 23 (304) |
|  |  |  |  |  |  |  | Median network, cingulum (258) |
| Right ventromedial prefrontal cortex | 6 | 50 | 2 | 1.227 | 0.00130 | 389 | Right superior frontal gyrus, medial, BA 10, 24, 32 (160) |
|  |  |  |  |  |  |  | Left anterior cingulate / paracingulate gyri, BA 24, 32 (187) |
|  |  |  |  |  |  |  | Left median network, cingulum (42) |
| **Patients with major depression disorder < healthy controls** | | | | | | | |
| Left gyrus rectus | -4 | 32 | -26 | -1.423 | 0.00029 | 943 | Left gyrus rectus, BA 11 (464) |
|  |  |  |  |  |  |  | Left superior frontal gyrus, medial/orbital part, BA 11 (230) |
|  |  |  |  |  |  |  | Right gyrus rectus, BA 11 (117) |
|  |  |  |  |  |  |  | Right superior frontal gyrus, medial/orbital, BA 11 （84） |
|  |  |  |  |  |  |  | Olfactory cortex, BA 11, 25 (48) |
| Right middle temporal gyrus | 46 | -72 | 10 | -1.139 | 0.00173 | 94 | Right middle temporal gyrus, BA 19, 37, 39 (82) |
|  |  |  |  |  |  |  | Right middle occipital gyrus, BA 19, 37, 39 (12) |
| Left superior frontal gyrus, orbital part | -16 | 58 | -8 | -1.192 | 0.00127 | 64 | Left superior frontal gyrus, orbital part, BA 11 (61) |
|  |  |  |  |  |  |  | Left middle frontal gyrus, orbital part, BA 11 (3) |
| Abbreviations: BA, Brodmann area; MNI, Montreal Neurological Institute; SDM, Seed-based d Mapping. | | | | | | | |

**Table S5. Results of the jackknife analysis in all included studies.**

| **Discarded study** | **Increased cortical thickness** | |  | **Decreased cortical thickness** | | |
| --- | --- | --- | --- | --- | --- | --- |
|  | Left PCC | Right vmPFC extending to left ACC |  | Left gyrus rectus | Right MTG | Left oSFG |
| Han[10] | Yes | Yes |  | Yes | Yes | Yes |
| Kakeda[11] | Yes | Yes |  | Yes | No | Yes |
| Lan[17] | Yes | Yes |  | Yes | Yes | Yes |
| Liu[12] | Yes | Yes |  | Yes | Yes | Yes |
| Na[13] | Yes | Yes |  | Yes | Yes | No |
| Niu[18] | Yes | Yes |  | Yes | Yes | Yes |
| Peng[14] | No | Yes |  | Yes | Yes | Yes |
| Qiu[15] | Yes | No |  | Yes | Yes | Yes |
| Späti[19] | Yes | Yes |  | Yes | Yes | Yes |
| Taylor[20] | Yes | Yes |  | Yes | Yes | Yes |
| Van[21] | No | Yes |  | No | Yes | Yes |
| Van[22] | Yes | Yes |  | Yes | Yes | Yes |
| Wagner[23] | Yes | Yes |  | Yes | Yes | Yes |
| Yang[16] | Yes | Yes |  | Yes | Yes | Yes |
| Zorlu[24] | Yes | Yes |  | Yes | Yes | Yes |
| **Total** | 13/15 | 14/15 |  | 14/15 | 14/15 | 14/15 |
| Abbreviations: ACC, anterior cingulate cortex; oSFG, orbital part of superior frontal gyrus; PCC, posterior cingulate cortex; vmPFC, ventromedial prefrontal cortex. | | | | | | |

**Table S6.** **Results of the jackknife analysis in studies with non-comorbid patients (12 studies).**

| **Discarded study** | **Increased cortical thickness** | |  | **Decreased cortical thickness** | | |
| --- | --- | --- | --- | --- | --- | --- |
|  | Left PCC | Right vmPFC extending to left ACC |  | Left gyrus rectus | Right MTG | Left oSFG |
| Han[10] | Yes | Yes |  | Yes | Yes | Yes |
| Kakeda[11] | Yes | Yes |  | Yes | No | Yes |
| Liu[12] | Yes | Yes |  | Yes | Yes | Yes |
| Na[13] | Yes | Yes |  | Yes | Yes | No |
| Niu[18] | Yes | Yes |  | Yes | Yes | Yes |
| Peng[14] | No | Yes |  | Yes | Yes | Yes |
| Qiu[15] | Yes | No |  | Yes | Yes | Yes |
| Taylor[20] | Yes | Yes |  | Yes | Yes | Yes |
| Van[21] | No | Yes |  | Yes | Yes | Yes |
| Van[22] | Yes | Yes |  | Yes | Yes | Yes |
| Wagner[23] | Yes | Yes |  | Yes | Yes | Yes |
| Zorlu[24] | Yes | Yes |  | Yes | Yes | Yes |
| **Total** | 10/12 | 11/12 |  | 12/12 | 11/12 | 11/12 |
| Abbreviations: ACC, anterior cingulate cortex; oSFG, orbital part of superior frontal gyrus; PCC, posterior cingulate cortex; vmPFC, ventromedial prefrontal cortex. | | | | | | |

**Table S7. Results of the jackknife analysis in studies with threshold correction (14 studies).**

| **Discarded study** | **Increased cortical thickness** | |  | **Decreased cortical thickness** | | |
| --- | --- | --- | --- | --- | --- | --- |
|  | Left PCC | Right vmPFC extending to left ACC |  | Left gyrus rectus | Right MTG | Left oSFG |
| Han[10] | Yes | Yes |  | Yes | Yes | Yes |
| Kakeda[11] | Yes | Yes |  | Yes | No | Yes |
| Lan[17] | Yes | Yes |  | Yes | Yes | Yes |
| Liu[12] | Yes | Yes |  | Yes | Yes | Yes |
| Na[13] | Yes | Yes |  | Yes | Yes | No |
| Niu[18] | Yes | Yes |  | Yes | Yes | Yes |
| Peng[14] | Yes | Yes |  | Yes | Yes | Yes |
| Qiu[15] | Yes | Yes |  | Yes | Yes | Yes |
| Späti[19] | Yes | Yes |  | Yes | Yes | Yes |
| Taylor[20] | Yes | Yes |  | Yes | Yes | Yes |
| Van[21] | No | Yes |  | Yes | Yes | Yes |
| Van[22] | Yes | Yes |  | Yes | Yes | Yes |
| Wagner[23] | Yes | Yes |  | Yes | Yes | Yes |
| Zorlu[24] | Yes | Yes |  | Yes | Yes | Yes |
| **Total** | 13/14 | 14/14 |  | 14/14 | 13/14 | 13/14 |
| Abbreviations: ACC, anterior cingulate cortex; oSFG, orbital part of superior frontal gyrus; PCC, posterior cingulate cortex; vmPFC, ventromedial prefrontal cortex. | | | | | | |

**Table S8. Differences in cortical thickness between medication-naive patients with MDD and HCs (7 studies [10-16]).**

| **Region** | **MNI coordinate** | | | **SDM** | ***P***, uncorrected | **Voxels** | **Cluster breakdown** (voxels) |
| --- | --- | --- | --- | --- | --- | --- | --- |
|  | x | y | z | ***Z* score** |  |  |  |
| **medication-naive patients with major depression disorder > healthy controls** | | | | | | | |
| Right middle frontal gyrus, orbital part | 36 | 54 | -2 | 1.158 | 0.00230 | 122 | Right middle frontal gyrus, orbital part, BA 10, 11, 47 (91) |
|  |  |  |  |  |  |  | Right superior frontal gyrus, orbital part, BA 10, 11 (31) |
| Right ventromedial prefrontal cortex | 4 | 54 | 2 | 1.156 | 0.00235 | 82 | Right superior frontal gyrus, medial, BA 10, 32 (68) |
|  |  |  |  |  |  |  | Right anterior cingulate / paracingulate gyri, BA 10, 32 (14) |
| **medication-naive patients with major depression disorder < healthy controls** | | | | | | | |
| Left superior frontal gyrus, orbital part | -18 | 62 | -4 | -1.306 | 0.00132 | 181 | Left superior frontal gyrus, orbital part, BA 11 (181) |
| Abbreviations: BA, Brodmann area; MNI, Montreal Neurological Institute; SDM, Seed-based d Mapping. | | | | | | | |

**Table S9. Results of the jackknife analysis in studies with medication-naive patients with MDD (7 studies).**

| **Discarded study** | **Increased cortical thickness** | |  | **Decreased cortical thickness** |
| --- | --- | --- | --- | --- |
|  | Right  oMFG | Right vmPFC |  | Left oSFG |
| Han[10] | Yes | Yes |  | Yes |
| Kakeda[11] | Yes | No |  | Yes |
| Liu[12] | Yes | No |  | Yes |
| Na[13] | Yes | Yes |  | No |
| Peng[14] | Yes | Yes |  | Yes |
| Qiu[15] | No | No |  | Yes |
| Yang[16] | Yes | Yes |  | Yes |
| **Total** | 6/7 | 4/7 |  | 6/7 |

Abbreviations: oMFG, orbital part of middle frontal gyrus; oSFG, orbital part of superior frontal gyrus;

vmPFC, ventromedial prefrontal cortex.

**Figure S1. Results of funnel plot analysis to test for publication bias**

**
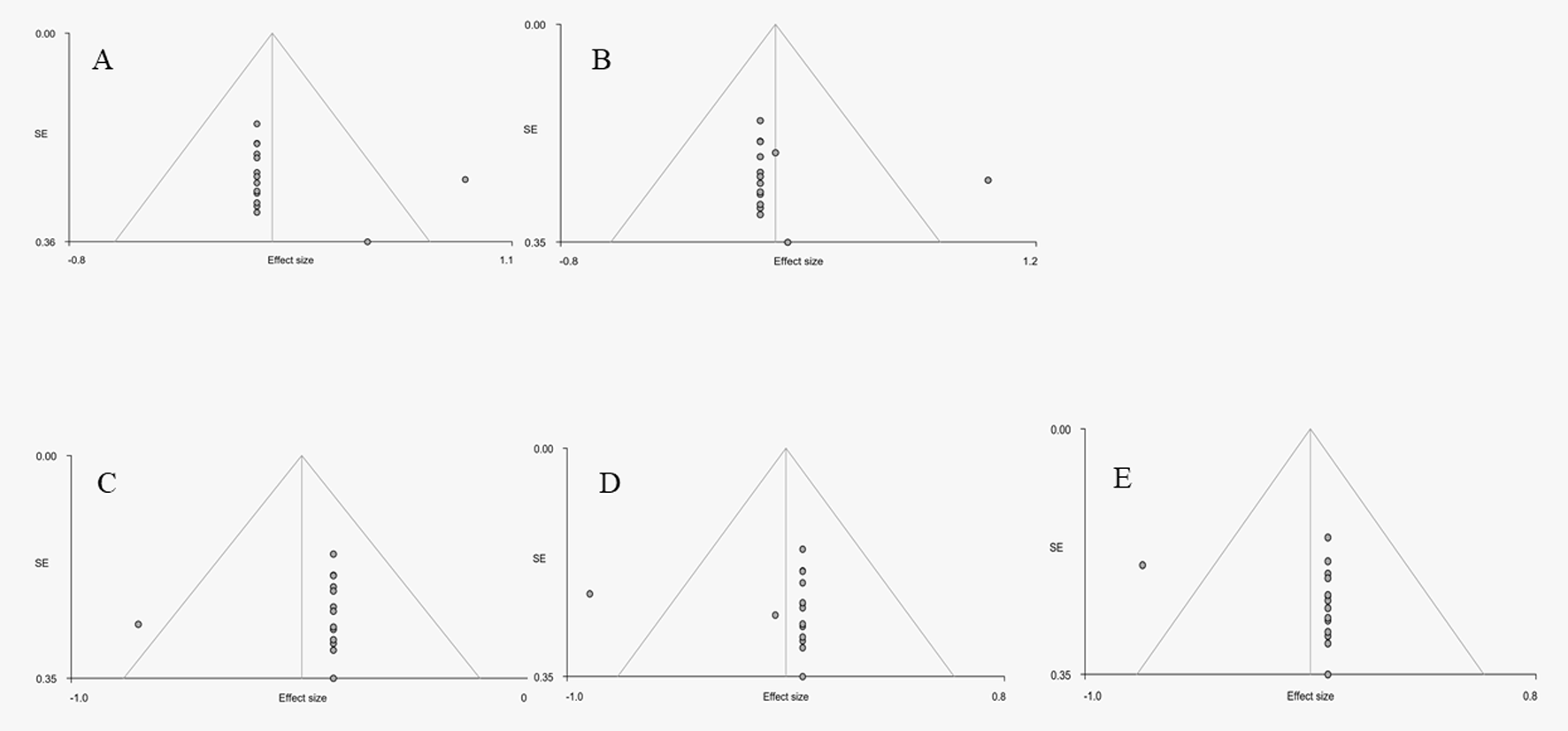
**

For the current pooled meta-analysis, the Egger’s test and funnel plots revealed no significant publication bias (A) in the left posterior cingulate cortex (*Z*=1.21, *t*=1.00, *df*=13, *P*=0.335), (B) in the right ventromedial prefrontal cortex (*Z*=−0.27, *t*=−0.21, *df*=13, *P*=0.834), (C) in the left gyrus rectus (*Z*=−1.41, *t*=−0.83, *df*=13, P=0.419), (D) in the right middle temporal gyrus (*Z*=0.02, *t*=0.01, *df*=13, *P*=0.989), and (E) in the left orbital part of superior frontal gyrus (*Z*=0.95, *t*=0.90, *df*=13, *P*=0.440) in medication-free patients with major depressive disorder than controls.

**Figure S2. Cortical thickness alterations in medication-naive patients with MDD than controls.**


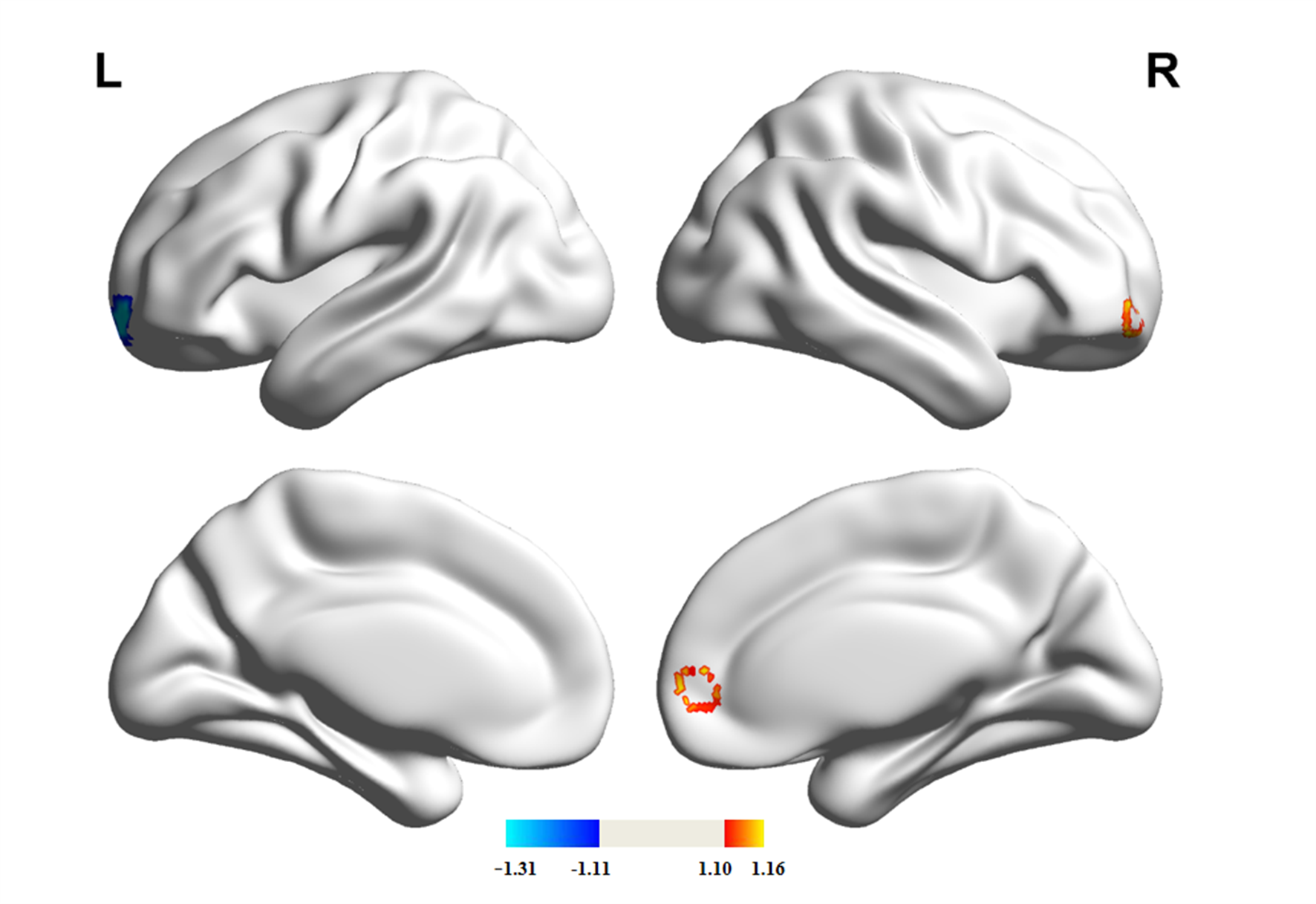


**References**

1 Knobloch K, Yoon U, Vogt PM. Preferred reporting items for systematic reviews and meta-analyses (PRISMA) statement and publication bias. Journal of cranio-maxillo-facial surgery : official publication of the European Association for Cranio-Maxillo-Facial Surgery. 2011;39(2):91-2.

2 Hasin DS, Goodwin RD, Stinson FS, Grant BF. Epidemiology of major depressive disorder: results from the National Epidemiologic Survey on Alcoholism and Related Conditions. Arch Gen Psychiatry. 2005;62(10):1097-106.

3 Mannan M, Mamun A, Doi S, Clavarino A. Prospective Associations between Depression and Obesity for Adolescent Males and Females- A Systematic Review and Meta-Analysis of Longitudinal Studies. PLoS one. 2016;11(6):e0157240.

4 Du M, Liu J, Chen Z, Huang X, Li J, Kuang W, et al. Brain gray matter volume alterations in late-life depression. Journal of psychiatry & neuroscience : JPN. 2014;39(6):397-406.

5 Radua J, Mataix-Cols D, Phillips ML, El-Hage W, Kronhaus DM, Cardoner N, et al. A new meta-analytic method for neuroimaging studies that combines reported peak coordinates and statistical parametric maps. European psychiatry : the journal of the Association of European Psychiatrists. 2012;27(8):605-11.

6 Radua J, Rubia K, Canales-Rodriguez EJ, Pomarol-Clotet E, Fusar-Poli P, Mataix-Cols D. Anisotropic kernels for coordinate-based meta-analyses of neuroimaging studies. Front Psychiatry. 2014;5:13.

7 Radua J, Mataix-Cols D. Voxel-wise meta-analysis of gray matter changes in obsessive-compulsive disorder. Br J Psychiatry. 2009;195(5):393-402.

8 Radua J, Grau M, van den Heuvel OA, Thiebaut de Schotten M, Stein DJ, Canales-Rodriguez EJ, et al. Multimodal voxel-based meta-analysis of white matter abnormalities in obsessive-compulsive disorder. Neuropsychopharmacology. 2014;39(7):1547-57.

9 Chen ZQ, Du MY, Zhao YJ, Huang XQ, Li J, Lui S, et al. Voxel-wise meta-analyses of brain blood flow and local synchrony abnormalities in medication-free patients with major depressive disorder. Journal of psychiatry & neuroscience : JPN. 2015;40(6):401-11.

10 Han K-M, Choi S, Jung J, Na K-S, Yoon H-K, Lee M-S, et al. Cortical thickness, cortical and subcortical volume, and white matter integrity in patients with their first episode of major depression. Journal of affective disorders. 2014;155:42-48.

11 Kakeda S, Watanabe K, Katsuki A, Sugimoto K, Igata N, Ueda I, et al. Relationship between interleukin (IL)-6 and brain morphology in drug-naive, first-episode major depressive disorder using surface-based morphometry. Sci Rep. 2018;8(1):10054.

12 Liu X, Kakeda S, Watanabe K, Yoshimura R, Abe O, Ide S, et al. Relationship between the cortical thickness and serum cortisol levels in drug-naive, first-episode patients with major depressive disorder: A surface-based morphometric study. Depression and anxiety. 2015;32(9):702-08.

13 Na K-S, Chang HS, Won E, Han K-M, Choi S, Tae WS, et al. Association between Glucocorticoid Receptor Methylation and Hippocampal Subfields in Major Depressive Disorder. PLoS one. 2014;9(1).

14 Peng D, Shi F, Li G, Fralick D, Shen T, Qiu M, et al. Surface vulnerability of cerebral cortex to major depressive disorder. PLoS one. 2015;10 (3) (no pagination)(e0120704).

15 Qiu L, Lui S, Kuang W, Huang X, Li J, Li J, et al. Regional increases of cortical thickness in untreated, first-episode major depressive disorder. Translational psychiatry. 2014;4:e378.

16 Yang X-h, Wang Y, Huang J, Zhu C-y, Liu X-q, Cheung EFC, et al. Increased prefrontal and parietal cortical thickness does not correlate with anhedonia in patients with untreated first-episode major depressive disorders. Psychiatry research Neuroimaging. 2015;234(1):144-51.

17 Lan MJ, Chhetry BT, Oquendo MA, Sublette ME, Sullivan G, Mann JJ, et al. Cortical thickness differences between bipolar depression and major depressive disorder. Bipolar disorders. 2014;16(4):378-88.

18 Niu M, Wang Y, Jia Y, Wang J, Zhong S, Lin J, et al. Common and Specific Abnormalities in Cortical Thickness in Patients with Major Depressive and Bipolar Disorders. EBioMedicine. 2017;16:162-71.

19 Späti J, Haenggi J, Doerig N, Ernst J, Sambataro F, Brakowski J, et al. Prefrontal Thinning Affects Functional Connectivity and Regional Homogeneity of the Anterior Cingulate Cortex in Depression. Neuropsychopharmacology. 2015;40(7):1640-48.

20 Taylor WD, Boyd B, McQuoid DR, Kudra K, Saleh A, MacFall JR. Widespread white matter but focal gray matter alterations in depressed individuals with thoughts of death. Progress in neuro-psychopharmacology & biological psychiatry. 2015;62:22-28.

21 van Eijndhoven P, van Wingen G, Katzenbauer M, Groen W, Tepest R, Fernandez G, et al. Paralimbic cortical thickness in first-episode depression: evidence for trait-related differences in mood regulation. The American journal of psychiatry. 2013;170(12):1477-86.

22 van Eijndhoven P, Mulders P, Kwekkeboom L, van Oostrom I, van Beek M, Janzing J, et al. Bilateral ECT induces bilateral increases in regional cortical thickness. Translational psychiatry. 2016;6(8):e874.

23 Wagner G, Schultz CC, Koch K, Schachtzabel C, Sauer H, Schlösser RG. Prefrontal cortical thickness in depressed patients with high-risk for suicidal behavior. Journal of psychiatric research. 2012;46(11):1449-55.

24 Zorlu N, Cropley VL, Zorlu PK, Delibas DH, Adibelli ZH, Baskin EP, et al. Effects of cigarette smoking on cortical thickness in major depressive disorder. Journal of psychiatric research. 2017;84:1-8.
